# miR-221 stimulates breast cancer cells and cancer-associated fibroblasts (CAFs) through selective interference with the A20/c-Rel/CTGF signaling

**DOI:** 10.1186/s13046-018-0767-6

**Published:** 2018-05-02

**Authors:** Maria Francesca Santolla, Rosamaria Lappano, Francesca Cirillo, Damiano Cosimo Rigiracciolo, Anna Sebastiani, Sergio Abonante, Pierfrancesco Tassone, Pierosandro Tagliaferri, Maria Teresa Di Martino, Marcello Maggiolini, Adele Vivacqua

**Affiliations:** 1 Department of Pharmacy, Health and Nutritional Sciences, University of Calabria, Rende, Italy; 2 Regional Hospital Cosenza, Cosenza, Italy; 3 Department of Experimental and Clinical Medicine, Magna Graecia University, Catanzaro, Italy

**Keywords:** Breast cancer, CAFs, miR-221, A20, C-Rel, CTGF

## Abstract

**Background:**

MicroRNA (miRNAs) are non-coding small RNA molecules that regulate gene expression by inhibiting the translation of target mRNAs. Among several dysregulated miRNAs in human cancer, the up-regulation of miR-221 has been associated with development of a variety of hematologic and solid malignancies. In this study, we investigated the involvement of miR-221 in breast cancer.

**Methods:**

TaqMan microRNA assay was used to detect the miR-221 levels in normal cells and in MDA-MB 231 and SkBr3 breast cancer cells as well as in main players of the tumor microenvironment, namely cancer-associated fibroblasts (CAFs). miR-221 mimic sequence and locked nucleic acid (LNA)-i-miR-221 construct were used to induce or inhibit, respectively, the miR-221 expression in cells used. Quantitative PCR and western blotting analysis were performed to evaluate the levels of the miR-221 target gene A20 (TNFAIP3), as well as the member of the NF-kB complex namely c-Rel and the connective tissue growth factor (CTGF). Chromatin immunoprecipitation (ChIP) assay was performed to ascertain the recruitment of c-Rel to the CTFG promoter. Finally, the cell growth and migration in the presence of LNA-i-miR-221 or silencing c-Rel and CTGF by specific short hairpin were assessed by cell count, colony formation and boyden chambers assays. Statistical analysis was performed by ANOVA.

**Results:**

We first demonstrated that LNA-i-miR-221 inhibits both endogenous and ectopic expression of miR-221 in our experimental models. Next, we found that the A20 down-regulation, as well as the up-regulation of c-Rel induced by miR-221 were no longer evident using LNA-i-miR-221. Moreover, we established that the miR-221 dependent recruitment of c-Rel to the NF-kB binding site located within the CTGF promoter region is prevented by using LNA-i-miR-221. Furthermore, we determined that the up-regulation of CTGF mRNA and protein levels by miR-221 is no longer evident using LNA-i-miR221 and silencing c-Rel. Finally, we assessed that cell growth and migration induced by miR-221 in MDA-MB 231 and SkBr3 breast cancer cells as well as in CAFs are abolished by LNAi-miR-221 and silencing c-Rel or CTGF.

**Conclusions:**

Overall, these data provide novel insights into the stimulatory action of miR-221 in breast cancer cells and CAFs, suggesting that its inhibition may be considered toward targeted therapeutic approaches in breast cancer patients.

## Background

microRNAs (miRNAs) are small non-coding RNA molecules (of ~ 22 nucleotides), which regulate the expression of up to 30% protein coding genes, usually binding to specific sites within the 3′ untranslated regions (3’-UTRs) of the mRNA targets [1]. The expression of miRNAs is cell- and tissue-specific, indicating that miRNAs are closely associated with cell differentiation and development [2]. In addition, miRNAs exert a regulatory role in several pathophysiological processes, including many types of tumor [2–5]. In this regard, it should be mentioned that a distinct miRNA may be found up-regulated in certain carcinomas and down-regulated in others, suggesting, therefore, a potential oncogenic and tumor suppressor function, respectively, depending on the cell context. Breast cancer, which represents the most common female malignancy in western countries [6], is one of the first solid tumors investigated for miRNA expression [7]. Among the most significant miRNAs overexpressed in breast carcinoma, miR-21 has been shown to mediate cell survival and invasion [7, 8]. Likewise, miR-144 was shown to induce stimulatory effects in breast cancer cells [9] and miR-103/miR-107 were associated with a poor outcome in patients affected by triple-negative breast cancer [10]. Next, the involvement of miR-221/miR-222 has been recently shown in many tumors [11]. For instance, miR-222 was implicated in the progression [12] and the drug-resistance [13] of breast cancer, whereas miR-221 elicited stimulatory effects in diverse types of malignancies down-regulating certain onco-suppressor genes [14, 15]. In addition, in bone marrow-derived macrophages, miR-221 was reported to down-regulate the expression of the ubiquitin-editing A20 [16] enzyme, which acts toward the maintenance of tissue homeostasis and the prevention of inflammatory disorders [17]. In this vein, it was demonstrated that A20 inhibits the activity of the nuclear factor kappa B (NF-kB) [18], which is largely involved in the development of many types of tumors [19, 20]. A variety of mechanisms may regulate the expression of A20, like specific A20 binding proteins (ABINs), the ubiquitin binding protein TAX1BP1 and the histone methyltransferase Ash1l [21–23]. In addition, miR-29c, miR-873 and let-7 have been reported to suppress A20 expression, therefore contributing to the activation of NF-kB signaling [24–26]. miR-125a and miR-125b were shown to also target A20 and aberrantly activate the NF-kB transduction pathway in B-cell lymphoma [27]. NF-kB embraces a family of transcription factors formed by hetero- or homo-dimers, including the subunits p65 (RelA) p50, p52, c-Rel or RelB [28]. The NF-kB inhibitor alpha (IkB)α, one of the members of the IkB family, keeps the dimers in an inactive form within the cytoplasm. The release from the IkBα leads to nuclear translocation of the NF-kB dimers and their DNA binding, hence leading to the transcription of a variety of genes [28]. Recently, it has been reported that A20 may inhibit the NF-kB activity by reducing the nuclear levels of c-Rel [29]. c-Rel is a 587–amino acid protein, which contains a highly conserved N-terminal DNA-binding/dimerization domain named Rel homology domain (RHD) [30]. The C-terminal half of c-Rel encompasses two C-terminal transactivation sequences (TAD1 and TAD2), separated from the RHD by a transactivation inhibitory domain (RID) [30]. c-Rel exists either as a homodimer or a heterodimer with p50, however c-Rel can also form dimers with p65 and p52 [30]. Dysregulated expression and activity of c-Rel have been demonstrated in various cancers [30]. In agreement with these findings, the ectopic expression of c-Rel triggered the development of breast tumors in transgenic mice [31]. It has been recently reported that NF-kB regulates the expression of the connective tissue growth factor (CTGF) [32, 33]. CTGF is involved in many cellular processes including cell adhesion, matrix production, structural remodelling, angiogenesis, cell proliferation and differentiation [34]. CTGF is mainly regulated by mechanical stresses as well as by a number of cytokines and growth factors [34]. Previous studies have reported its involvement in various malignancies as breast and endometrial tumors [35], melanoma [36], gastric [37], pancreatic [38], prostate [39], hepatic [40] and colon cancer [41].

On the basis of the aforementioned observations, we attempted to provide novel insights into the molecular mechanism through which miR-221 may induce oncogenic effects in breast tumor, using as experimental models crucial players of the tumor microenvironment as cancer-associated fibroblasts (CAFs) [42], the MDA-MB 231 and SkBr3 breast cancer cells. We found that miR-221 down-regulates A20 expression and increases both c-Rel and CTGF, leading to cell growth and migration. Worthy, these effects were abrogated silencing c-Rel and CTGF expression and using the specific Locked Nucleic Acid (LNA)-Inhibitor of miR-221 (LNA-i-miR-221), which is a 13-mer oligonucleotide designed specifically to sequester the miR-221 [43, 44]. In particular, the LNA-i-miR-221, recently approved for first-in-human clinical trial (EudraCT 201,700,261,533), takes advantage from both LNA technology and phosphorothioate backbone to increase the seed sequence binding and nuclease resistance in vivo, respectively [45, 46]. Together, our data highlight the oncogenic action of miR-221 in CAFs and breast cancer cells, hence suggesting that its inhibition may represent a further preventive and therapeutic strategy in breast cancer.

## Methods

### Bioinformatic tools

The putative promoter sequences of CTGF (− 625 bp/+ 62 bp) and A20 3’-UTR were retrieved from the National Center for Biotechnology Information (NCBI) (http://www.ncbi.nlm.nih.gov). Prediction of transcription factors within CTGF promoter was performed using TransFac (http://www.generegulation.com) site and Promo 3.0.2 (http://alggen.lsi.upc.es/). miR-221 target genes were identified using miRNAbase (http://www.miRNAbase.org), Targetscan (http://www.targetscan.org) and miRDip (http://ophid.utoronto.ca/mirDIP/) sites.

### Cell cultures

The breast cancer cell lines (triple-negative MDA-MB 231 and overexpressing epidermal growth factor receptor 2 SkBr3) and non-malignant breast epithelial cell (MCF10A) were obtained from the ATCC (Manassas, USA). SkBr3 and MDA-MB 231 breast cancer cells were maintained, respectively, in RPMI-1640 without phenol red and DMEM/F12 media (Life Technologies, Milan, Italy) with a supplement of 10% fetal bovine serum (FBS) and 100 μg/ml of penicillin/streptomycin (Life Technologies, Milan, Italy). MCF10A cell line were cultured in DMEM/F12 media (Life Technologies, Milan, Italy) following the instruction of ATCC (Manassas, USA). All cell types were used less than 6 months after resuscitation and routinely tested and authenticated according to the ATCC suggestions. CAFs were extracted from invasive mammary ductal carcinomas obtained from mastectomies, while normal fibroblasts were isolated from a non-cancerous breast tissue at least 2 cm from the outer tumor margin. Briefly, in both cases specimens were cut into smaller pieces (1–2 mm diameter), placed in digestion solution (400 IU collagenase I, 100 IU hyaluronidase, and 10% FBS, containing antibiotic and antimycotic solution) and incubated overnight at 37 °C. The cells were then separated by differential centrifugation at 90×g for 2 min. Supernatant containing fibroblasts was centrifuged at 485×g for 8 min; the pellet obtained was suspended in fibroblasts growth medium (Medium 199 and Ham’s F12 mixed 1:1 and supplemented with 10% FBS) and cultured at 37 °C in 5% CO_2_. Primary cells cultures of breast fibroblasts were characterized by immunofluorescence. Briefly cells were incubated with human anti-vimentin (V9, sc-6260) and human anti-cytokeratin 14 (LL001 sc-53,253), both from Santa Cruz Biotechnology (DBA, Milan, Italy) (data not shown). To characterize fibroblasts activation, we used anti-fibroblast activated protein α (FAPα) antibody (SS-13, sc-100,528; Santa Cruz Biotechnology, DBA, Milan, Italy) (data not shown). Signed informed consent from all the patients was obtained and all samples were collected, identified and used in accordance with approval by the Institutional Ethical Committee Board (Regional Hospital, Cosenza, Italy). All cell lines were grown in a 37 °C incubator with 5% CO_2_.

### Plasmid and transfection

The mimic miR-221 (miR-221) and negative control (miR-Ctrl) sequences were purchased from Ambion (Life Technologies, Milan, Italy). Custom LNA oligonucleotides were provided by Exiqon (Vedbaek, DenMark). LNA-i-miR-221 is a 13-mer DNA/LNA oligonucleotide whose sequence is CAGACAATGTAGC, with a fully PS-modified backbone. It was purified by HPLC followed by Na + salt exchange and lyophilisation [45]. The CTGF luciferase reporter plasmid p(− 1999/+ 36)-Luc (CTGF-luc), based on the backbone of vector pGL3-basic (Promega) [47], was a gift from Dr. B. Chaqour. c-Rel shRNA sequence (shRel) was obtained from TRC consortium (TRCN0000039986) and cloned, as described previously [48], in the piggyBac transposon vector (PB-H1-shRNA-GFP), kindly provided by Dr. W.T. Khaled, University of Cambridge, UK. The shRNA construct for CTGF, obtained from the Open Biosystems (www.Biocat.de), contains the clone ID TRCN0000061950 and is based on the lentiviral expression vector pLKO.1.

### RNA extraction and real time-PCR

Cells were maintained in regular growth medium and then switched to medium lacking serum before the transfection with 25 nM miR-Ctrl, miR-221, 100 nM LNA-i-miR-221 and 5 μg short hairpin plasmids, as indicated. Total RNA were extracted from cultured cells using miRVana Isolation Kit (Ambion, Life Technologies, Milan, Italy) according to the manufacturer’s recommendations. The RNA concentrations were determined using Gene5 2.01 Software in Synergy H1 Hybrid Multi-Mode Microplate Reader (BioTek, AHSI, Milan Italy). cDNA for miRNA expression was synthesized from 100 ng of total RNA using the TaqMan microRNA Reverse Transcription Kit (Applied Biosystems, Life Technologies, Milan, Italy) and the expression levels of miR-221 were quantified by TaqMan microRNA Assay Kit (Applied Biosystems, Life Technologies, Milan, Italy). Real-time PCR analysis for mature miR-221 was performed using the primers for the internal control RNU6B (assay ID 001093) and miR-221 (assay ID 000524). In order to measure the mRNA levels of A20 and CTGF, 2 μg of total RNA were reversely transcribed using the murine leukaemia virus reverse transcriptase (Life Technologies, Milan, Italy), as indicated by the manufacturer. The quantitative PCR was performed using SYBR Green PCR Master Mix (Applied Biosystems, Life Technologies, Milan, Italy). Specific primers for Actin, which was used as internal control, A20 and CTGF genes were designed using Primer Express version 2.0 software (Applied Biosystems Inc., Milano, Italy). The sequences were as follows: Actin Fwd: 5’-AAGCCAACCCCACTTCTCTCTAA-3′ and Rev.: 5’-CACCTCCCCTGTGTGGACTT-3′; A20 Fwd: 5’-CTTGTGGCGCTGAAAACGAA-3′ and Rev.: 5’-CTGAACGCCCCACATGTACT-3′; CTGF Fwd: 5’-GGCCCAGACCCAACTATGATT-3′ and Rev.: 5’-GAACAGGCGCTCCACTCTGT-3′. All experiments were performed in triplicate using QuantStudio 6&7 Flex Real Time PCR System (Applied Biosystems, Life Technologies, Milan, Italy). The data were normalized to the geometric mean of housekeeping gene to control the variability into expression levels and fold changes were calculated by relative quantification compared to respective scrambled controls.

### Western blotting

Cells were maintained in complete medium before the transfection assays, which are performed in medium without serum for 48 h and then lysed in RIPA buffer containing a mixture of protease inhibitors. Equal amounts of protein extract were resolved on SDS-polyacrylamide gel, transferred to a nitrocellulose membrane (Amersham Biosciences, Italy), probed overnight at 4 °C with antibodies against: A20 (A-12, sc-166,692) and β-Actin (AC-15, sc-69,879) (Santa Cruz Biotechnology, DBA, Italy), CTGF (Origene, DBA, Milan, Italy), c-Rel (Cell Signaling Technology, Milan, Italy) and then revealed using the ECL™ Western Blotting Analysis System (GE Healthcare, Italy).

### Chromatin ImmunoPrecipitation (ChIP) assays

The day before ChIP analysis, cells were shifted to medium lacking serum and then transfected for 48 h with miR-Ctrl and 25 nM miR-221 alone or in presence of 100 nM LNA-i-miR-221. ChIP assay was performed as previously described [35]. In brief, the immune cleared chromatin was immunoprecipitated with anti c-Rel or non specific IgG (Santa Cruz Biotecnology, DBA), used as negative control. A 4 μl volume of each sample and input DNA was used as template to amplify, by real-time PCR a region containing a NF-kB binding site located within the promoter region of CTGF. The primer sequences were: 5’-ACGGAGGAATGCTGAGTGTC-3′ (forward) and 5’-GGCGGCCGAGGCTTTTATAC-3′ (reverse). Real-time PCR data were normalized respect to unprocessed lysates (Input) and the results were reported as fold changes respect to non specific IgG.

### Luciferase assays

Cells were seeded in regular growth medium into 24-well plates. The next day the growth medium was replaced with medium lacking serum and the transfection was performed using X-tremeGene9 reagent, as recommended by the manufacturer (Roche Diagnostics), with a mixture containing CTGF-luc, the internal control pRL-TK, miR-Ctrl or miR-221, alone or in presence of LNA-i-miR-221, shRNA or shRel, alone or in combination with miR-221. Luciferase activity was measured after 48 h using the Dual Luciferase kit (Promega, Milan, Italy) according to the manufacturer’s instructions. Firefly luciferase values were normalized to the internal transfection control provided by the Renilla luciferase activity. The normalized relative light unit (RLU) values obtained from cells transfected with respective scrambled controls were set as 1-fold induction upon which the activity induced by miR-221 was calculated.

### Cell proliferation assays

For quantitative proliferation assay, cells (1 × 10^4^) were seeded in 24-well plates in regular growth medium. Cells were washed, once they had attached, and then incubated in medium containing 2.5% charcoal stripped fetal bovine serum, before the transfection with 25 nM miR-221, 100 nM LNA-i-miR-221 and 500 ng of the indicated short hairpins. After 48 h the medium was changed and cell transfection was renewed. Evaluation of cell growth was performed on day 6 using automatic counter (Countess™-Invitrogen).

### Colony formation assays

For colony formation assays, cells were transfected as indicated, and then seeded into 6-well plates (2.5 × 10^4^ or 5 × 10^4^ cells per well). The transfections were renewed every 2 days during the assay. After 10 days of incubation, cells were washed with PBS, fixed in acetone:methanol (1:1) for 3 min at room temperature and then stained with 0.5% crystal violet in 20% methanol for 5 min or Giemsa for 10 min. Pictures were captured by using a digital camera. Colonies, with over 50 cells, were counted using the program WCIF ImageJ for Windows.

### Migration assays

Migration assays were performed by using boyden chambers (Costar Transwell, 8 mm polycarbonate membrane, Sigma Aldrich, Milan, Italy). Cells were transfected with 25 nM miR-221, 100 nM LNA-i-miR-221 and 500 ng/well shRel or shCTGF as indicated, in medium without serum for 48 h and then seeded in the upper chambers. In the bottom of the chambers was added regular medium. 8 h after seeding the cells on the bottom side of the membrane were fixed, stained with Giemsa and counted by using Cytation 3 Cell Imaging Multimode Reader (BioTek, Winooski, VT).

### Statistical analysis

Data were analysed by one-way ANOVA with Dunnett’s multiple comparisons where applicable, using GraphPad Prism version 6.01 (GraphPad Software, Inc., San Diego, CA, USA). *p* < 0.05 (*) and *p* < 0.01 (**) were considered statistically significant.

## Results

### miR-221 down-regulates A20 expression in CAFs, MDA-MB 231 and SkBr3 breast cancer cells

**Fig. 1:**
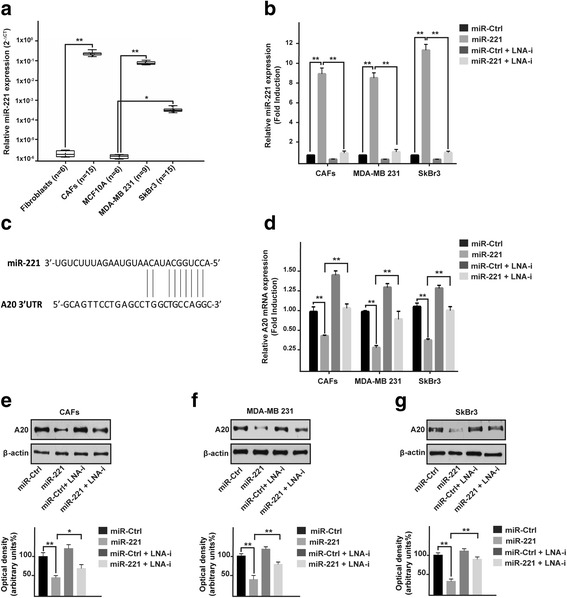
miR-221 regulates A20 expression in CAFs and breast cancer cells. **a** Expression of miR-221 in fibroblasts, CAFs, MCF10A, MDA-MB 231 and SkBr3 cells. Raw Ct data were normalized to the housekeeping RNU6 levels and expressed as ΔCt values using the comparative cross threshold (Ct) method. **b** miR-221 expression in CAFs, MDA-MB 231 and SkBr3 breast cancer cells after transfection for 48 h with 25 nM miR-Ctrl and 25 nM miR-221, alone or in combination with 100 nM LNA-i-miR-221 (LNA-i). **c** Schematic alignment between the miR-221 sequence and the 3’-UTR mRNA region of A20. **d** A20 mRNA expression in CAFs, MDA-MB 231 and SkBr3 cells transfected for 48 h with 25 nM miR-Ctrl and 25 nM miR-221, alone or in combination with 100 nM LNA-i-miR-221 (LNA-i). A20 protein expression in CAFs (**e**), MDA-MB 231 (**f**) and SkBr3 (**g**) cells transfected for 48 h with 25 nM miR-Ctrl and 25 nM miR-221, alone or in in combination with 100 nM LNA-i-miR-221 (LNA-i); β-actin serves as a loading control. Below panels show densitometric analysis of the blots normalized to the loading controls. Each column represents the mean ± SD of three independent experiments performed in triplicate. The data are shown as fold induction respect to cells transfected with miR-Ctrl. (*) indicates *p* < 0.05 and (**) *p* < 0.01

On the basis of previous studies showing that miR-221 may act as an oncogenic factor in certain malignancies (reviewed in [11]), we began the present investigation assessing that the levels of the endogenous miR-221 are higher in crucial players of the tumor microenvironment as CAFs and in both MDA-MB 231 and SkBr3 breast cancer cells respect to normal fibroblasts and non-transformed MCF10A breast cells (Fig. 1a). In order to provide novel insights into the mechanisms through which miR-221 elicits a tumorigenic action, we then ascertained that the miR-221 inhibitor named LNA-i-miR-221 effectively reduces the levels of miR-221, which was ectopically expressed in CAFs, MDA-MB 231 and SkBr3 cells (Fig. 1b). Using available bioinformatics tools (http://www.microrna.org; http://www.targetscan.org; http://ophid.utoronto.ca/mirDIP/), we identified a putative miR-221 binding site in 3’-UTR region of the ubiquitin-editing enzyme TNFAIP3, namely A20 (Fig. 1c), recently also demonstrated through luciferase assay in macrophages by Zhao et al. [16]. In accordance with these findings, we ascertained that the ectopic expression of miR-221 lowers mRNA and protein levels of A20 in CAFs, MDA-MB 231 and SkBr3 cells (Fig. 1d-g), however these effects were no longer evident in the presence of LNA-i-miR-221 (Fig. 1d-g). Altogether, our results point to the ability of miR-221 in regulating A20 expression in our model system.

### miR-221 prompts the expression of c-Rel and its recruitment to the CTGF promoter region in CAFs, MDA-MB 231 and SkBr3 breast cancer cells

**Fig. 2:**
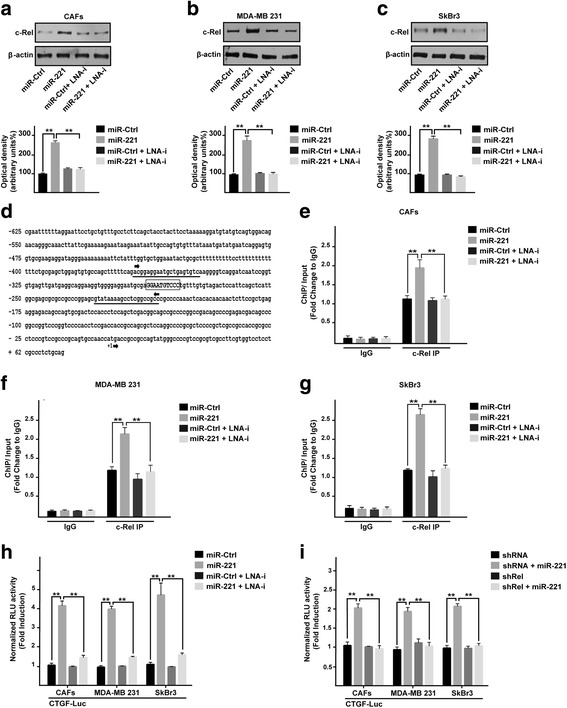
miR-221 prompts the protein expression of c-Rel and its recruitment to the CTGF promoter region. c-Rel protein expression in CAFs (**a**), MDA-MB 231 (**b**) and SkBr3 (**c**) cells transfected for 48 h with 25 nM miR-Ctrl and 25 nM miR-221, alone or in combination with 100 nM LNA-i-miR-221 (LNA-i). β-actin serves as a loading control. Below panels show densitometric analysis of the blots normalized to the loading controls. **d** Putative NF-kB binding site (capital letters in the rectangle) located within the CTGF promoter sequence (− 625 bp to + 62 bp). The transcriptional start site is indicated as + 1. The position of primers used for ChIP-qPCR analyses is underlined. Recruitment of c-Rel to the NF-kB binding site within the CTGF promoter sequence in CAFs (**e**), MDA-MB 231 (**f**) and SkBr3 (**g**) cells transfected for 48 h with 25 nM miR-Ctrl and 25 nM miR-221, alone or in combination with 100 nM LNA-i-miR-221 (LNA-i). Data were normalized to the Input and reported as fold changes respect to IgG. **h** Luciferase activity of the CTGF reporter gene in CAFs, MDA-MB 231 and SkBr3 cells transfected for 48 h with 25 nM miR-Ctrl and 25 nM miR-221, alone or in combination with 100 nM LNA-i-miR-221 (LNA-i). **i** Luciferase activity of the CTGF reporter gene in CAFs, MDA-MB 231 and SkBr3 cells transfected for 8 h with shRNA or shRel and then transfected for 48 h with 25 nM miR-221. The luciferase activity was normalized to the internal transfection control; values of cells receiving scrambled controls were set as 1-fold induction upon which the activity obtained with the indicated effectors was calculated. Each column represents the mean ± SD of three independent experiments performed in triplicate. (**) indicates *p* < 0.01

**Fig. 3:**
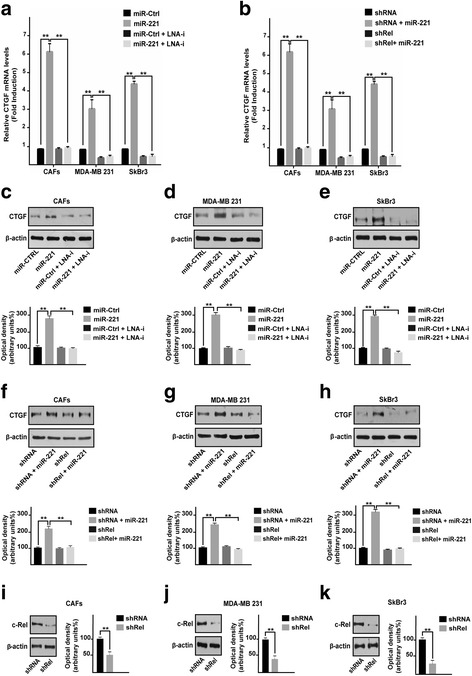
c-Rel is involved in the up-regulation of the CTGF triggered by miR-221. **a** CTGF mRNA levels in CAFs, MDA-MB 231 and SkBr3 cells transfected for 48 h with 25 nM miR-Ctrl and 25 nM miR-221, alone or in combination with 100 nM LNA-i-miR-221 (LNA-i). Data are shown as fold changes respect to the scrambled controls. **b** CTGF mRNA levels in CAFs, MDA-MB 231 and SkBr3 cells transfected for 8 h with shRNA or shRel and then transfected for 48 h with 25 nM miR-221. Data are shown as fold changes respect to the scrambled controls. CTGF protein levels in CAFs (**c**), MDA-MB 231 (**d**) and SkBr3 (**e**) cells transfected for 48 h with 25 nM miR-Ctrl and 25 nM miR-221, alone or in combination with 100 nM LNA-i-miR-221 (LNA-i). β-actin serves as a loading control. Below panels show densitometric analysis of the blots normalized to the loading controls. CTGF protein levels in CAFs (**f**), MDA-MB 231 (**g**) and SkBr3 (**h**) cells transfected for 8 h with shRNA or shRel and then transfected for 48 h with 25 nM miR-221. β-actin serves as a loading control. Below panels show densitometric analysis of the blots normalized to the loading controls. Efficacy of c-Rel silencing in CAFs (**i**), MDA-MB 231 (**j**) and SkBr3 (**k**) cells. β-actin serves as a loading control. Side panels show densitometric analysis of the blots normalized to the loading controls. Results shown are representative of at least three independent experiments. (**) indicates *p* < 0.01

Previous studies have shown that A20 may regulate NF-kB, in particular a negative correlation between the levels of A20 and the expression of a NF-kB component named c-Rel has been reported [29]. In this vein, we found that the miR-221 dependent c-Rel protein expression was no longer evident in the presence of LNA-i-miR-221 in CAFs, MDA-MB 231 and SkBr3 cells (Fig. 2a-c). As NF-kB activity has been demonstrated to regulate CTGF expression [32, 33], we analyzed the CTGF promoter sequence (http://www.ncbi.nlm.nih.gov; http://alggen.lsi.upc.es/; http://www.generegulation.com) identifying a putative NF-kB binding site (Fig. 2d). Worthy, performing a ChIP analysis we assessed that the recruitment of c-Rel to the CTGF promoter sequence induced by miR-221 is abrogated in the presence of LNA-i-miR-221 (Fig. 2e-g). Moreover and in agreement with these results, the transactivation of a CTGF-luc reporter construct by miR-221 was repressed by LNA-i-miR-221 (Fig. 2h) as well as by shRNA silencing of c-Rel expression (Fig. 2i). In accordance with these results, the up-regulation of CTGF by miR-221 at both mRNA and protein levels was abolished by LNA-i-miR-221 (Fig. 3a, c-e) or c-Rel knock-down (Fig. 3b, f-k). Collectively, these data suggest that c-Rel is involved in the up-regulation of CTGF expression by miR-221 in CAFs, MDA-MB 231 and SkBr3 cells.

### miR-221 induces growth and migratory effects through c-Rel and CTGF in CAFs, MDA-MB 231 and SkBr3 breast cancer cells

**Fig. 4:**
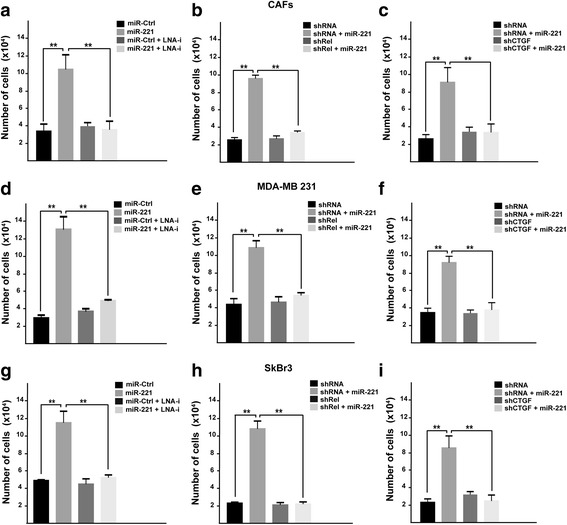
miR-221 induces proliferative effects in CAFs, MDA-MB 231 and SkBr3 cells. Cell proliferation in (**a**) CAFs, (**d**) MDA-MB 231 and (**g**) SkBr3 cells transfected every 2 days with 25 nM miR-Ctrl and 25 nM miR-221, alone or in combination with 100 nM LNA-i-miR-221 (LNA-i) and then counted on day 6. Cell proliferation in CAFs (**b**) MDA-MB 231 (**e**) and SkBr3 (**h**) cells transfected with shRNA or shRel for 8 h and then transfected for 48 h with 25 nM miR-221. Cell proliferation in CAFs (**c**), MDA-MB 231 (**f**) and SkBr3 (**i**) cells transfected with shRNA or shCTGF for 8 h and then transfected for 48 h with 25 nM miR-221. The transfections were renewed every 2 days and cells were counted on day 6. Each data point is the mean ± SD of three independent experiments performed in triplicate. (**) indicates *p* < 0.01

**Fig. 5:**
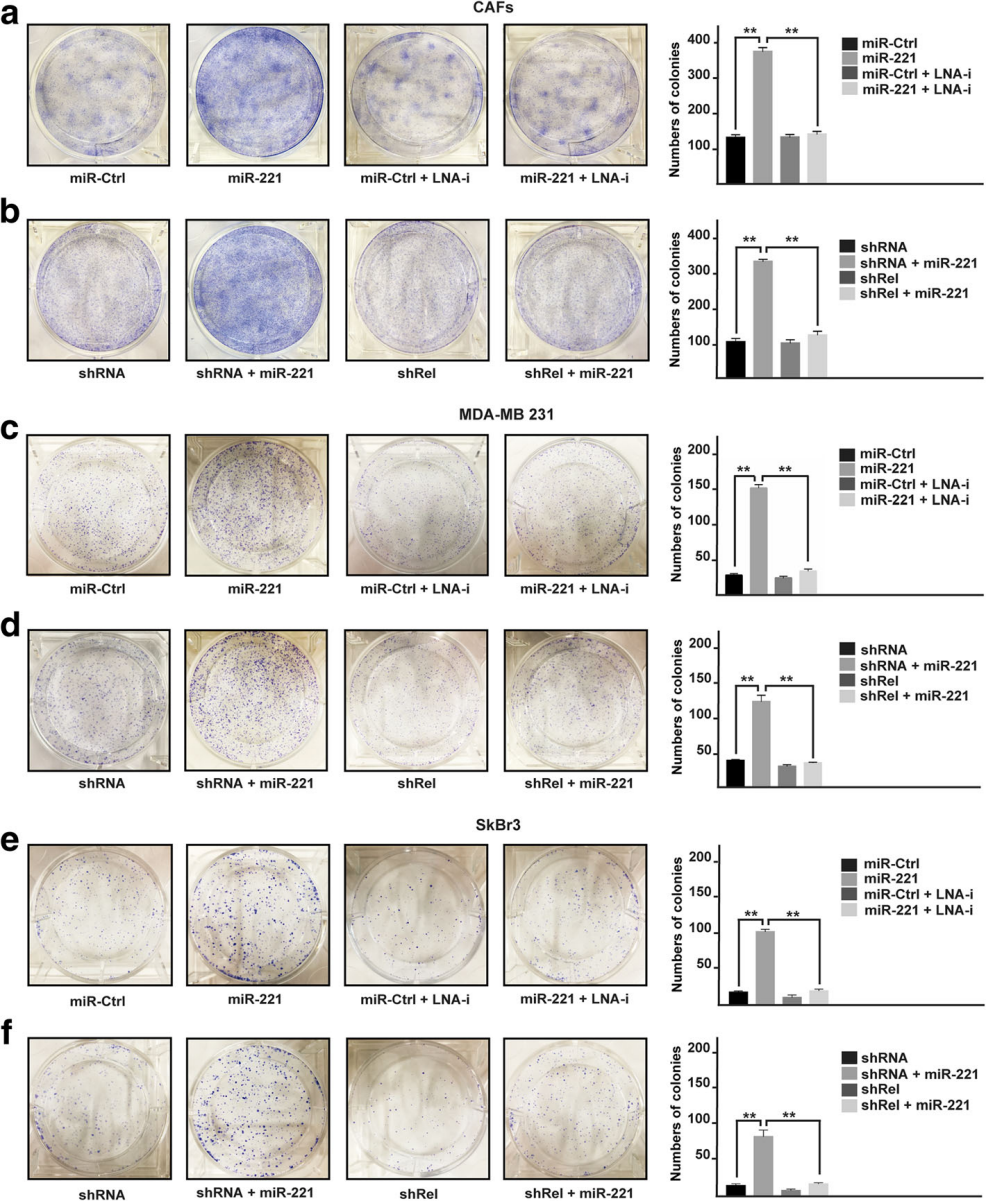
miR-221 promotes colony formation in CAFs, MDA-MB 231 and SkBr3 cells. Colony formation in CAFs (**a**, **b**), MDA-MB 231 (**c**, **d**) and SkBr3 (**e**, **f**) cells transfected every 2 days as indicated, after 10 days of incubation cell colonies were stained and pictures were captured by a digital camera. Colonies were counted using the program WCIF ImageJ for Windows. Each data point is the mean ± SD of three independent experiments performed in triplicate. (**) indicates *p* < 0.01

**Fig. 6:**
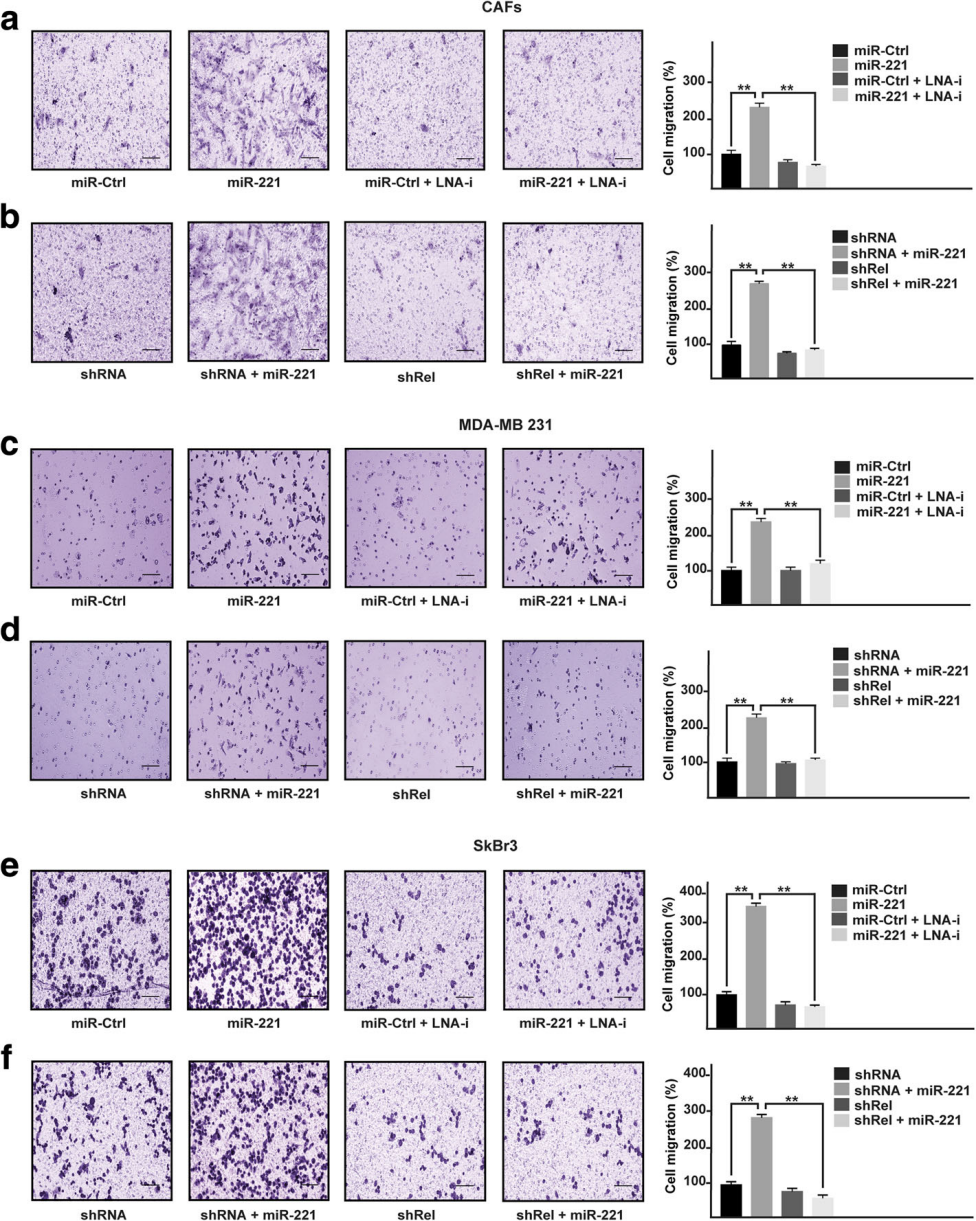
miR-221 triggers migratory effects in CAFs, MDA-MB 231 and SkBr3 cells. Cell migration as evaluated by Boyden Chamber assay in (**a**) CAFs, (**c**) MDA-MB 231 and (**e**) SkBr3 cells transfected for 48 h with 25 nM miR-Ctrl and 25 nM miR-221, alone or in combination with 100 nM LNA-i-miR-221 (LNA-i). Cell migration, evaluated by Boyden Chamber assay, in CAFs (**b**) MDA-MB 231 (**d**) and SkBr3 (**f**) cells transfected with shRNA or shRel for 8 h and then transfected for 48 h with 25 nM miR-221. Cells were counted in at least 10 random fields at 10× magnification (Scale bar =200 μm) in three independent experiments performed in triplicate. (**) indicates *p* < 0.01

As a biological counterpart of the results described above, we ascertained that the proliferative responses elicited by miR-221 are prevented in the presence of LNA-i-miR-221 (Fig. 4a, d, g), or by c-Rel silencing (Fig. 4b, e, h) and CTGF induced expression (Fig. 4c, f, i) in CAFs, MDA-MB 231 and SkBr3 cells. By colony formation assay, we then assessed that the clonogenic capacity induced by miR-221 in CAFs, MDA-MB 231 and SkBr3 is no longer evident in the presence of LNA-i-miR-221 (Fig. 5a, c, e), or by shRNA mediated silencing of c-Rel (Fig. 5b, d, f) or by CTGF expression (data not shown). Performing Boyden chamber assay in CAFs, MDA-MB-231 and SkBr3 cells, we also determined that the migratory effects stimulated by miR-221 are abolished using LNA-i-miR-221 (Fig. 6a, c, e), knocking-down c-Rel (Fig. 6b, d, f) or CTGF expression (data not shown). Overall, these results indicate that both c-Rel and CTGF are involved in the proliferative and migratory effects triggered by miR-221 in our model system.

## Discussion

In the present study, we have ascertained novel molecular mechanisms by which miR-221 may exert an oncogenic action in breast cancer. In particular, we have shown that miR-221 could trigger proliferative and migratory effects through the interference with A20/c-Rel/CTGF signaling in main players of the tumor microenvironment as CAFs, in MDA-MB 231 and SkBr3 breast cancer cells. Worthy, these biological responses were prevented using the synthetic inhibitor of miR-221 namely LNA-i-miR-221 or silencing c-Rel and CTGF expression. Overall, our data provide novel evidence on the oncogenic role of miR-221, hence suggesting the usefulness of its inhibition in further preventive and therapeutic strategies against breast tumor.

Breast cancer is a heterogeneous disease that includes various subtypes, with different biological behaviour and clinical outcome [49]. Based on the gene expression profile, three major subtypes of breast cancer have been identified: the luminal (A and B), the basal-like and the epidermal growth factor receptor 2 overexpressing type [49]. The breast cancer subtype expressing hormone receptors, usually identified as luminal A and B, have a favourable prognosis undergoing anti-hormone treatments [50]. Tumors overexpressing the epidermal growth factor receptor 2 are treated with anti HER2 monoclonal antibodies as trastuzumab and pertuzumab or with immunoconjugates as TDM1 or kinase inhibitors as lapatinib or neratinib, mostly combined [51], whereas the triple-negative breast cancer that is treated with conventional and moderately successful chemotherapies displays a poor prognosis and a high risk of relapse [52, 53]. Presently, there is increasing evidence that components of the tumor microenvironment, as CAFs, may play a main role in cancer progression and invasiveness [42, 54]. The growth characteristics of CAFs are different from those of fibroblasts associated with normal breast epithelial cells [55]. In particular, CAFs associated with invasive breast carcinoma cells show an abnormal migratory behaviour in vitro [55], altered levels of growth factors like CTGF [56] and insulin-like growth factors I and II [57], increased expression of inflammatory genes [58]. In addition, several studies have recently suggested that every cellular process is likely regulated by miRNAs and aberrant miRNAs expression may play a pathogenic role in several diseases, including cancer [7, 59]. Among dysregulated miRNAs, miR-221 is of relevant interest since it is strongly upregulated in a variety of hematologic and solid malignancies, including breast cancer (reviewed in [11]). In particular, increased levels of miR-221, which form a cluster together with miR-222 within the human chromosome X, have been related to the invasion of breast cancer cells and advanced clinical stages in breast tumor patients [60]. Hence, targeting miR-221 by specific inhibitors like LNA-i-miR-221 may represent a new promising strategy to overcome cancer progression, as in breast tumor [42–45].

In this scenario, we provide a better understanding of the molecular mechanism through which miR-221 may be involved in the progression of breast cancer. First, we have found that in CAFs, in MDA-MB 231 and SkBr3 breast cancer cells, miR-221 down-regulates the expression of the ubiquitin-editing enzyme, A20, at both mRNA and protein levels. These results are consistent with previous data obtained in other cell contexts, showing that miR-221 directly targets the 3’-UTR region of A20 [16]. A20 is a Cys2/Cys2 zinc finger protein, which is induced by a variety of inflammatory stimuli and acts as a negative regulator of NF-kB [16, 18]. Of note, NF-kB signaling is tightly controlled by ubiquitination and A20, through its de-ubiquitinating activity, is one of the proteins that affects this process. For instance, it has been suggested that A20 induces the ubiquitination of the para-caspase MALT1, thus preventing the formation of the MALT1-IkB kinase complex and the consequent activation of the NF-kB signaling that mainly involves the heterodimers RelA, c-Rel, and p50 [29]. In agreement with these observations, we also found that miR-221 induces c-Rel expression, therefore suggesting that miR-221 may regulate NF-kB action. Interacting with other components of the NF-kB complex, c-Rel forms dimers that binding to specific sequences in the promoter region of target genes modulate gene expression [30]. By bioinformatic analysis, we found a putative NF-kB binding site located within the CTGF promoter sequence and we demonstrated that miR-221 induces the recruitment of c-Rel within the CTGF promoter region, toward an increase of CTGF expression. Corroborating these findings, we finally assessed that the growth and migratory effects induced by miR-221 in CAFs, MDA-MB 231 and SkBr3 cells, are prevented by LNA-i-miR-221 or by silencing of c-Rel and CTGF expression.

## Conclusions

In summary, our data show that miR-221 elicits a stimulatory action not only in breast cancer cells but also in main component of the tumor microenvironment like CAFs, through the involvement of A20/c-Rel/CTGF signaling. Consequently, the inhibition of miR-221 by LNA-i-miR-221 may be considered in novel therapeutic approaches in breast cancer.

## Abbreviations

3’-UTRs: 3′ untranslated regions
CAFs: Cancer Associated Fibroblasts
CTGF: Connective Tissue Growth Factor
LNA-i-miR-221: Locked Nucleic Acid (LNA)-i-miR-221
NF-kB: Nuclear factor kappa B
TNFAIP3: Tumor necrosis factor alpha-induced protein 3
